# Acridinium 3-carb­oxy­pyrazine-2-carboxyl­ate

**DOI:** 10.1107/S1600536810030588

**Published:** 2010-08-11

**Authors:** Jafar Attar Gharamaleki, Zohreh Derikvand, Helen Stoeckli-Evans

**Affiliations:** a Young Researchers Club, Islamic Azad University, North Tehran Branch, Tehran, Iran; b Department of Chemistry, Faculty of Science, Islamic Azad University, Khorramabad Branch, Khorramabad, Iran; c Institut de Physique, Universite de Neuchâtel, Rue Emile-Argand 11, CP 158, CH-2009 Neuchâtel, Switzerland

## Abstract

The title ion pair, C_13_H_10_N^+^·C_6_H_3_N_2_O_4_^−^, contains a protonated acridine cation and a 3-carb­oxy­pyrazine-2-carboxyl­ate monoanion, which are linked together through O—H⋯O, N—H⋯O and weak C—H⋯O hydrogen bonds. These hydrogen bonds generate a *C*(10) chain graph-set motif. The crystal structure is further stabilized by extensive π–π stacking inter­actions between nearly parallel [dihedral angle = 1.21(2)°] acridine systems. The shortest distance between the centroids of the six-membered rings within the cations is 3.6315 (8) Å. In addition, C—H⋯π edge-to-face inter­actions are present.

## Related literature

For the biological activity of acridines, see: Talacki *et al.* (1974 ▶); Achenson (1956 ▶); Fan *et al.* (1997 ▶); Bandoli *et al.* (1994 ▶). For ion pairs reported from pyrazine-2,3-dicarb­oxy­lic acid, pz-2,3-dcH_2_, with various organic bases such as 8-hy­droxy­quinoline and guanidine, see: Smith *et al.* (2006*a* ▶,*b* ▶). For a recently reported proton-transfer compound of acridine and benzene-1,3,5-tricarb­oxy­lic acid, see: Derikvand *et al.* (2009 ▶). For graph-set analysis, see: Bernstein *et al.* (1995 ▶).
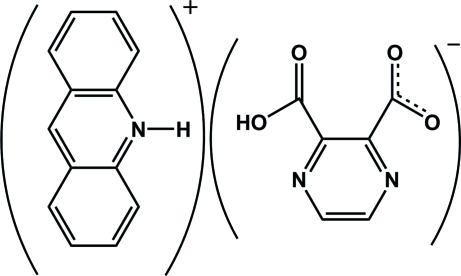


## Experimental

### 

#### Crystal data

C_13_H_10_N^+^·C_6_H_3_N_2_O_4_^−^
*M* *_r_* = 347.32
Orthorhombic, 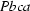

*a* = 10.0597 (9) Å
*b* = 15.0623 (12) Å
*c* = 20.306 (2) Å
*V* = 3076.8 (5) Å^3^
*Z* = 8
Mo *K*α radiation
μ = 0.11 mm^−1^
*T* = 223 K
0.45 × 0.36 × 0.25 mm

#### Data collection

Stoe IPDS 2 diffractometer
11459 measured reflections
4070 independent reflections
2639 reflections with *I* > 2σ(*I*)
*R*_int_ = 0.037

#### Refinement

*R*[*F* ^2^ > 2σ(*F* ^2^)] = 0.036
*wR*(*F* ^2^) = 0.080
*S* = 0.88
4070 reflections
244 parameters
H atoms treated by a mixture of independent and constrained refinement
Δρ_max_ = 0.26 e Å^−3^
Δρ_min_ = −0.15 e Å^−3^

### 

Data collection: *X-AREA* (Stoe & Cie, 2006 ▶); cell refinement: *X-AREA*; data reduction: *X-RED32* (Stoe & Cie, 2006 ▶); program(s) used to solve structure: *SHELXS97* (Sheldrick, 2008 ▶); program(s) used to refine structure: *SHELXL97* (Sheldrick, 2008 ▶); molecular graphics: *PLATON* (Spek, 2009 ▶); software used to prepare material for publication: *SHELXL97*.

## Supplementary Material

Crystal structure: contains datablocks I, global. DOI: 10.1107/S1600536810030588/om2349sup1.cif

Structure factors: contains datablocks I. DOI: 10.1107/S1600536810030588/om2349Isup2.hkl

Additional supplementary materials:  crystallographic information; 3D view; checkCIF report

## Figures and Tables

**Table 1:** Hydrogen-bond geometry (Å, °) *Cg*1 is the centroid of the N1,N2,C1–C4 ring.

*D*—H⋯*A*	*D*—H	H⋯*A*	*D*⋯*A*	*D*—H⋯*A*
O1—H1⋯O3^i^	0.948 (17)	1.628 (17)	2.5736 (13)	174.7 (16)
N3—H3*N*⋯O3^ii^	0.944 (16)	1.709 (16)	2.6374 (13)	167.0 (13)
C10—H10⋯O2	0.94	2.50	3.1896 (17)	131
C18—H18⋯*Cg*1^iii^	0.94	2.95	3.7213 (16)	140

Symmetry codes: (i) 
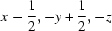
; (ii) 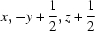; (iii) 
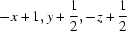
.

**Fig. 1.**
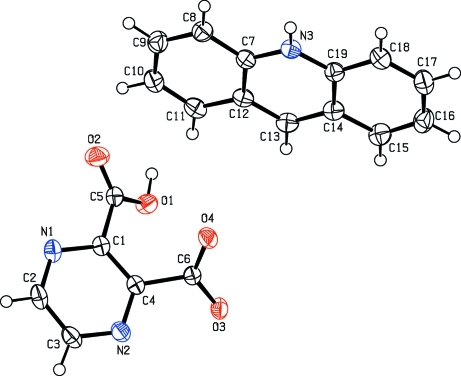
Molecular structure of the title ion pair, with ellipsoids drawn at the 50% probability level.

**Fig. 2.**
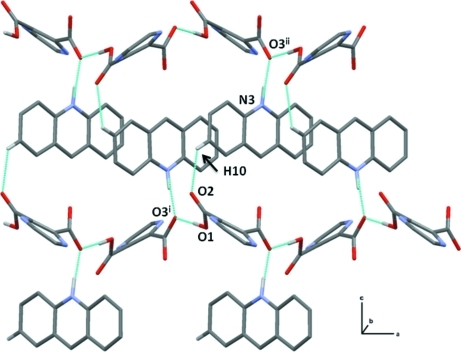
The O–H···O hydrogen bonds link (pz-2,3-dcH)- units into chains with C(10) chain graph-set motifs and N–H···O and C–H···O hydrogen bonds link (acrH)+ cations to these chains

**Fig. 3.**
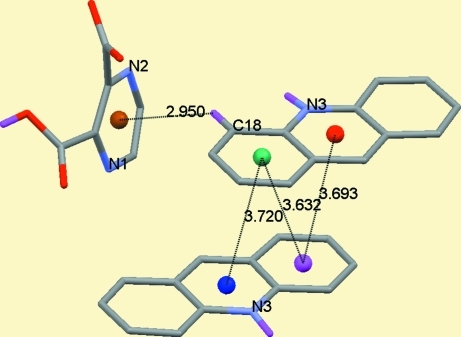
C—H···π and π–π stacking interactions between C13H10N+.C6H3N2O4- fragments.

**Fig. 4.**
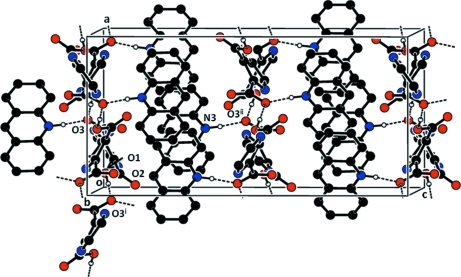
A view along the b-axis of the crystal packing of the title compound. Hydrogen atoms not involved in the N—H···N and O—H···O hydrogen bonds (dashed lines) have been omitted for clarity (see Table 1 for details).

## supplementary crystallographic information

### Comment

Acridine is structurally related to anthracene wherein one of the central CH groups is replaced by nitrogen. Acridines are found to have a wide range of biological activity, such as mutagenic, antitumor (Talacki *et al.*, 1974) and antibacterial (Achenson, 1956) properties.The ability of acridine to interact with DNA is also established (Fan *et al.*, 1997). In addition, acridine compounds are considered to be efficient drugs for the treatment of Alzheimer's disease (Bandoli *et al.*, 1994). Pyrazine-2,3-dicarboxylic acid, pz-2,3-dcH_2_, has proved to be well suited for the construction of the multi-dimensional frameworks due to the presence of two adjacent carboxylic acid groups.

There have been several attempts to prepare proton transfer compounds involving carboxylic acids and amines. For example, ion pairs have been reported between pz-2,3-dcH_2_ and various organic bases such as 8-hydroxy quinoline (Smith *et al.*, 2006*a*) and guanidine (Smith *et al.*, 2006*b*). The crystal structure of a proton-transfer compound of acridine and benzene-1,3,5-tricarboxylic acid has been reported (Derikvand *et al.*, 2009). In this work, we report a new proton transfer compound obtained from pyrazine-2,3-dicarboxylic acid as a proton donor and acridine as an acceptor.

The molecular structure of the title compound (Fig. 1), confirmed the full proton transfer, *i.e.* protonation of the acridine N atom and deprotonation of one of the carboxylic acid groups in the pyrazine-2,3-dicarboxylic acid. The carboxylate groups of the anion are twisted by 46.09 (7) and 37.34 (7)° with respect to the aromatic ring of pyrazine.

Non-covalent interactions cause the structure to form a self-assembled system. A hydrogen bonded motif is found involving the anion and cation fragments. The (pz-2,3-dcH)^-^ units are linked to each other by O–H···O hydrogen bonds to form one-dimensional chains with a C(10) graph-set motif (Bernstein *et al.*, 1995). In contrast, the N–H···O and C–H···O hydrogen bonds link the (acrH)^+^ cations to these chains (Fig. 2, Table 1). In addition, interactions consisting of π–π stacking with centroid-to-centroid distances of 3.6315 (8) to 3.7202 (9) Å between two acridine parallel rings are also present. Furthermore, C–H···π edge-to-face interactions are present involving the CH group of acridine with an aromatic ring of (pz-2,3-dcH)^-^, with a H···π distance of 2.95 Å for C18–H18···*Cg*1^i^ [symmetry code: (i) 1 - *x*, 1/2 + *y*, 1/2 - *z*; *Cg*1= centroid of ring N1,N2,C1–C3; Fig. 3)]. The sum of these weak non-covalent interactions seems to play an important role in the crystal packing. The unit cell packing diagram of the title compound, showing the N—H···O and O—H···O hydrogen bonding, is shown in Fig. 4 and details are given in Table 1.

### Experimental

The reaction between a solution of pyrazine-2,3-dicarboxylic acid (160 mg, 1 mmol) in 20 ml water and acridine (180 mg, 1 mmol) in 10 ml methanol, in a 1:1 molar ratio, gave brown rod-like crystals after slow evaporation of the solvent at room temperature.

### Refinement

The OH and NH H-atoms were located in a difference electron-density map and were freely refined: O—H = 0.948 (7) Å, N—H = 0.944 (16) Å. The C-bound H-atoms were included in calculated positions and treated as riding atoms: C—H = 0.94 Å with *U*_iso_(H) = 1.2*U*_eq_(parent C-atom).

### Crystal data

**Table:**

C_13_H_10_N^+^·C_6_H_3_N_2_O_4_^−^	*F*(000) = 1440
*M**_r_* = 347.32	*D*_x_ = 1.500 Mg m^−^^3^
Orthorhombic, *P**b**c**a*	Mo *K*α radiation, λ = 0.71073 Å
Hall symbol: -P 2ac 2ab	Cell parameters from 6855 reflections
*a* = 10.0597 (9) Å	θ = 1.7–29.6°
*b* = 15.0623 (12) Å	µ = 0.11 mm^−^^1^
*c* = 20.306 (2) Å	*T* = 223 K
*V* = 3076.8 (5) Å^3^	Rod, brown
*Z* = 8	0.45 × 0.36 × 0.25 mm

### Data collection

**Table:**

Stoe IPDS 2 diffractometer	2639 reflections with *I* > 2σ(*I*)
Radiation source: fine-focus sealed tube	*R*_int_ = 0.037
graphite	θ_max_ = 29.2°, θ_min_ = 2.6°
Detector resolution: 6.67 pixels mm^-1^	*h* = −13→8
φ and ω scans	*k* = −20→19
11459 measured reflections	*l* = −27→19
4070 independent reflections	

### Refinement

**Table:**

Refinement on *F*^2^	Secondary atom site location: difference Fourier map
Least-squares matrix: full	Hydrogen site location: inferred from neighbouring sites
*R*[*F*^2^ > 2σ(*F*^2^)] = 0.036	H atoms treated by a mixture of independent and constrained refinement
*wR*(*F*^2^) = 0.080	*w* = 1/[σ^2^(*F*_o_^2^) + (0.0423*P*)^2^] where *P* = (*F*_o_^2^ + 2*F*_c_^2^)/3
*S* = 0.88	(Δ/σ)_max_ = 0.001
4070 reflections	Δρ_max_ = 0.26 e Å^−^^3^
244 parameters	Δρ_min_ = −0.15 e Å^−^^3^
0 restraints	Extinction correction: *SHELXL97* (Sheldrick, 2008), Fc^*^=kFc[1+0.001xFc^2^λ^3^/sin(2θ)]^-1/4^
Primary atom site location: structure-invariant direct methods	Extinction coefficient: 0.0053 (5)

### Special details

**Table:**

Geometry. Bond distances, angles *etc*. have been calculated using the rounded fractional coordinates. All su's are estimated from the variances of the (full) variance-covariance matrix. The cell e.s.d.'s are taken into account in the estimation of distances, angles and torsion angles
Refinement. Refinement of *F*^2^ against ALL reflections. The weighted *R*-factor *wR* and goodness of fit *S* are based on *F*^2^, conventional *R*-factors *R* are based on *F*, with *F* set to zero for negative *F*^2^. The threshold expression of *F*^2^ > σ(*F*^2^) is used only for calculating *R*-factors(gt) *etc*. and is not relevant to the choice of reflections for refinement. *R*-factors based on *F*^2^ are statistically about twice as large as those based on *F*, and *R*- factors based on ALL data will be even larger.

### Fractional atomic coordinates and isotropic or equivalent isotropic displacement parameters (Å^2^)

**Table:**

	*x*	*y*	*z*	*U*_iso_*/*U*_eq_	
N3	0.39070 (12)	0.36166 (6)	0.33546 (5)	0.0262 (3)	
C7	0.30038 (14)	0.30865 (7)	0.30586 (6)	0.0258 (3)	
C8	0.20976 (15)	0.25926 (7)	0.34397 (6)	0.0307 (4)	
C9	0.11849 (16)	0.20748 (8)	0.31306 (7)	0.0337 (4)	
C10	0.11455 (15)	0.20109 (7)	0.24330 (7)	0.0330 (4)	
C11	0.20060 (14)	0.24823 (8)	0.20582 (7)	0.0315 (4)	
C12	0.29589 (14)	0.30485 (7)	0.23570 (6)	0.0268 (3)	
C13	0.38426 (15)	0.35715 (7)	0.20033 (6)	0.0291 (4)	
C14	0.47339 (14)	0.41345 (7)	0.23175 (6)	0.0273 (3)	
C15	0.56395 (15)	0.46933 (8)	0.19741 (7)	0.0334 (4)	
C16	0.64831 (16)	0.52222 (8)	0.23146 (7)	0.0375 (4)	
C17	0.64816 (16)	0.52281 (8)	0.30109 (7)	0.0377 (4)	
C18	0.56445 (15)	0.47022 (8)	0.33632 (7)	0.0326 (4)	
C19	0.47527 (14)	0.41474 (7)	0.30188 (6)	0.0266 (3)	
O1	0.09781 (10)	0.19372 (5)	0.01540 (5)	0.0310 (3)	
O2	0.02145 (11)	0.12332 (6)	0.10467 (5)	0.0394 (3)	
O3	0.43276 (10)	0.18006 (5)	−0.04013 (4)	0.0292 (3)	
O4	0.37854 (10)	0.18308 (5)	0.06640 (4)	0.0321 (3)	
N1	0.12751 (12)	−0.03103 (6)	0.04271 (5)	0.0297 (3)	
N2	0.35933 (12)	−0.00112 (6)	−0.02910 (5)	0.0294 (3)	
C1	0.17714 (13)	0.05048 (7)	0.03435 (6)	0.0233 (3)	
C2	0.19595 (15)	−0.09731 (7)	0.01534 (7)	0.0320 (4)	
C3	0.30534 (15)	−0.08191 (7)	−0.02325 (7)	0.0323 (4)	
C4	0.29838 (13)	0.06421 (7)	0.00344 (5)	0.0224 (3)	
C5	0.09064 (13)	0.12624 (7)	0.05662 (6)	0.0248 (3)	
C6	0.37460 (13)	0.15038 (7)	0.01113 (6)	0.0229 (3)	
H3N	0.4009 (17)	0.3551 (9)	0.3814 (8)	0.040 (4)*	
H8	0.21240	0.26210	0.39020	0.0370*	
H9	0.05680	0.17530	0.33830	0.0400*	
H10	0.05170	0.16390	0.22300	0.0400*	
H11	0.19720	0.24340	0.15970	0.0380*	
H13	0.38380	0.35440	0.15410	0.0350*	
H15	0.56520	0.46950	0.15110	0.0400*	
H16	0.70780	0.55910	0.20850	0.0450*	
H17	0.70730	0.56050	0.32350	0.0450*	
H18	0.56590	0.47080	0.38260	0.0390*	
H1	0.0346 (19)	0.2380 (10)	0.0263 (8)	0.051 (5)*	
H2	0.16840	−0.15610	0.02270	0.0380*	
H3	0.34360	−0.12960	−0.04630	0.0390*	

### Atomic displacement parameters (Å^2^)

**Table:**

	*U*^11^	*U*^22^	*U*^33^	*U*^12^	*U*^13^	*U*^23^
N3	0.0286 (6)	0.0282 (5)	0.0218 (5)	0.0012 (4)	−0.0019 (5)	0.0008 (4)
C7	0.0276 (7)	0.0236 (5)	0.0262 (6)	0.0030 (5)	−0.0019 (6)	0.0002 (4)
C8	0.0344 (8)	0.0307 (6)	0.0270 (6)	−0.0001 (5)	0.0011 (6)	0.0019 (5)
C9	0.0338 (8)	0.0288 (6)	0.0385 (7)	−0.0013 (6)	0.0026 (6)	0.0029 (5)
C10	0.0312 (8)	0.0274 (5)	0.0403 (7)	−0.0006 (5)	−0.0052 (7)	−0.0038 (5)
C11	0.0347 (8)	0.0306 (6)	0.0293 (6)	0.0043 (6)	−0.0046 (6)	−0.0047 (5)
C12	0.0290 (7)	0.0264 (5)	0.0249 (6)	0.0051 (5)	−0.0016 (6)	−0.0004 (4)
C13	0.0327 (8)	0.0316 (6)	0.0230 (6)	0.0053 (5)	−0.0010 (6)	0.0023 (5)
C14	0.0286 (7)	0.0263 (5)	0.0271 (6)	0.0055 (5)	−0.0014 (6)	0.0035 (5)
C15	0.0383 (9)	0.0304 (6)	0.0316 (7)	0.0037 (6)	0.0023 (6)	0.0087 (5)
C16	0.0364 (9)	0.0298 (6)	0.0464 (8)	−0.0021 (6)	0.0033 (7)	0.0089 (6)
C17	0.0366 (9)	0.0289 (6)	0.0475 (8)	−0.0035 (6)	−0.0036 (7)	−0.0011 (5)
C18	0.0356 (8)	0.0302 (6)	0.0320 (7)	0.0001 (6)	−0.0038 (6)	−0.0021 (5)
C19	0.0273 (7)	0.0237 (5)	0.0288 (6)	0.0036 (5)	−0.0008 (6)	0.0016 (5)
O1	0.0317 (6)	0.0247 (4)	0.0365 (5)	0.0077 (4)	0.0062 (4)	0.0064 (3)
O2	0.0492 (7)	0.0356 (4)	0.0333 (5)	0.0027 (5)	0.0135 (5)	0.0015 (4)
O3	0.0357 (6)	0.0276 (4)	0.0244 (4)	−0.0102 (4)	0.0000 (4)	0.0008 (3)
O4	0.0382 (6)	0.0310 (4)	0.0271 (4)	−0.0074 (4)	−0.0005 (4)	−0.0061 (3)
N1	0.0293 (6)	0.0233 (5)	0.0364 (6)	−0.0043 (4)	−0.0031 (5)	0.0027 (4)
N2	0.0288 (6)	0.0261 (5)	0.0334 (6)	−0.0002 (4)	−0.0018 (5)	−0.0049 (4)
C1	0.0251 (7)	0.0219 (5)	0.0229 (6)	−0.0011 (5)	−0.0039 (5)	0.0016 (4)
C2	0.0325 (8)	0.0192 (5)	0.0442 (7)	−0.0035 (5)	−0.0090 (7)	0.0000 (5)
C3	0.0328 (8)	0.0233 (5)	0.0407 (8)	0.0024 (5)	−0.0059 (6)	−0.0080 (5)
C4	0.0253 (7)	0.0210 (5)	0.0209 (5)	0.0002 (4)	−0.0042 (5)	0.0009 (4)
C5	0.0248 (7)	0.0237 (5)	0.0259 (6)	−0.0029 (5)	−0.0020 (5)	0.0003 (4)
C6	0.0207 (7)	0.0216 (5)	0.0263 (6)	0.0006 (4)	−0.0026 (5)	0.0005 (4)

### Geometric parameters (Å, °)

**Table:**

O1—C5	1.3187 (14)	C14—C15	1.4229 (19)
O2—C5	1.1993 (16)	C15—C16	1.354 (2)
O3—C6	1.2750 (15)	C16—C17	1.414 (2)
O4—C6	1.2263 (14)	C17—C18	1.360 (2)
O1—H1	0.948 (17)	C18—C19	1.4115 (19)
N3—C7	1.3507 (17)	C8—H8	0.9400
N3—C19	1.3520 (16)	C9—H9	0.9400
N3—H3N	0.944 (16)	C10—H10	0.9400
N1—C2	1.3340 (16)	C11—H11	0.9400
N1—C1	1.3362 (15)	C13—H13	0.9400
N2—C4	1.3345 (15)	C15—H15	0.9400
N2—C3	1.3379 (15)	C16—H16	0.9400
C7—C8	1.4083 (18)	C17—H17	0.9400
C7—C12	1.4265 (17)	C18—H18	0.9400
C8—C9	1.358 (2)	C1—C4	1.3872 (18)
C9—C10	1.420 (2)	C1—C5	1.5046 (16)
C10—C11	1.3538 (19)	C2—C3	1.371 (2)
C11—C12	1.4193 (18)	C4—C6	1.5156 (16)
C12—C13	1.3881 (18)	C2—H2	0.9400
C13—C14	1.3893 (18)	C3—H3	0.9400
C14—C19	1.4243 (17)		
			
C5—O1—H1	111.0 (10)	C11—C10—H10	120.00
C7—N3—C19	123.26 (11)	C12—C11—H11	120.00
C19—N3—H3N	119.5 (10)	C10—C11—H11	120.00
C7—N3—H3N	116.8 (9)	C12—C13—H13	119.00
C1—N1—C2	116.22 (11)	C14—C13—H13	119.00
C3—N2—C4	116.12 (11)	C14—C15—H15	120.00
N3—C7—C12	119.30 (11)	C16—C15—H15	120.00
C8—C7—C12	120.47 (11)	C17—C16—H16	120.00
N3—C7—C8	120.21 (11)	C15—C16—H16	120.00
C7—C8—C9	119.14 (12)	C16—C17—H17	119.00
C8—C9—C10	121.24 (13)	C18—C17—H17	119.00
C9—C10—C11	120.48 (13)	C17—C18—H18	121.00
C10—C11—C12	120.44 (13)	C19—C18—H18	121.00
C11—C12—C13	123.53 (12)	N1—C1—C5	116.27 (11)
C7—C12—C13	118.28 (11)	C4—C1—C5	122.10 (10)
C7—C12—C11	118.19 (12)	N1—C1—C4	121.53 (11)
C12—C13—C14	121.48 (11)	N1—C2—C3	121.73 (10)
C13—C14—C19	118.43 (11)	N2—C3—C2	122.05 (11)
C15—C14—C19	118.26 (11)	N2—C4—C1	121.20 (10)
C13—C14—C15	123.31 (12)	N2—C4—C6	116.75 (11)
C14—C15—C16	119.94 (13)	C1—C4—C6	121.73 (10)
C15—C16—C17	120.91 (13)	O1—C5—C1	111.23 (10)
C16—C17—C18	121.55 (13)	O2—C5—C1	123.53 (10)
C17—C18—C19	118.55 (13)	O1—C5—O2	125.20 (11)
N3—C19—C18	120.01 (11)	O3—C6—C4	116.64 (10)
N3—C19—C14	119.20 (11)	O4—C6—C4	117.04 (10)
C14—C19—C18	120.79 (12)	O3—C6—O4	126.26 (11)
C7—C8—H8	120.00	N1—C2—H2	119.00
C9—C8—H8	120.00	C3—C2—H2	119.00
C8—C9—H9	119.00	N2—C3—H3	119.00
C10—C9—H9	119.00	C2—C3—H3	119.00
C9—C10—H10	120.00		
			
C19—N3—C7—C8	−176.00 (12)	C13—C14—C19—N3	−0.13 (18)
C19—N3—C7—C12	2.58 (18)	C13—C14—C19—C18	179.56 (12)
C7—N3—C19—C14	−2.11 (18)	C13—C14—C15—C16	−179.74 (13)
C7—N3—C19—C18	178.21 (12)	C19—C14—C15—C16	−0.39 (19)
C1—N1—C2—C3	−4.7 (2)	C15—C14—C19—N3	−179.52 (11)
C2—N1—C1—C4	−4.93 (18)	C15—C14—C19—C18	0.17 (18)
C2—N1—C1—C5	171.56 (11)	C14—C15—C16—C17	0.1 (2)
C3—N2—C4—C6	165.73 (11)	C15—C16—C17—C18	0.4 (2)
C3—N2—C4—C1	−7.87 (17)	C16—C17—C18—C19	−0.6 (2)
C4—N2—C3—C2	−1.7 (2)	C17—C18—C19—C14	0.33 (19)
N3—C7—C12—C11	179.63 (11)	C17—C18—C19—N3	−179.97 (13)
N3—C7—C12—C13	−0.81 (18)	N1—C1—C4—N2	11.70 (18)
C12—C7—C8—C9	0.41 (19)	N1—C1—C4—C6	−161.58 (11)
N3—C7—C8—C9	178.98 (12)	C5—C1—C4—N2	−164.58 (11)
C8—C7—C12—C11	−1.79 (18)	C5—C1—C4—C6	22.15 (17)
C8—C7—C12—C13	177.77 (12)	N1—C1—C5—O1	−140.94 (11)
C7—C8—C9—C10	1.2 (2)	N1—C1—C5—O2	36.83 (18)
C8—C9—C10—C11	−1.4 (2)	C4—C1—C5—O1	35.52 (16)
C9—C10—C11—C12	−0.1 (2)	C4—C1—C5—O2	−146.71 (13)
C10—C11—C12—C13	−177.91 (12)	N1—C2—C3—N2	8.4 (2)
C10—C11—C12—C7	1.62 (19)	N2—C4—C6—O3	45.62 (15)
C11—C12—C13—C14	178.19 (12)	N2—C4—C6—O4	−131.77 (12)
C7—C12—C13—C14	−1.35 (19)	C1—C4—C6—O3	−140.82 (12)
C12—C13—C14—C15	−178.83 (12)	C1—C4—C6—O4	41.78 (17)
C12—C13—C14—C19	1.81 (19)		

### Hydrogen-bond geometry (Å, °)

**Table:**

*Cg*1 is the centroid of the N1,N2,C1–C4 ring.

**Table:**

*D*—H···*A*	*D*—H	H···*A*	*D*···*A*	*D*—H···*A*
O1—H1···O3^i^	0.948 (17)	1.628 (17)	2.5736 (13)	174.7 (16)
N3—H3N···O3^ii^	0.944 (16)	1.709 (16)	2.6374 (13)	167.0 (13)
C10—H10···O2	0.94	2.50	3.1896 (17)	131
C18—H18···Cg1^iii^	0.94	2.95	3.7213 (16)	140

Symmetry codes: (i) *x*−1/2, −*y*+1/2, −*z*; (ii) *x*, −*y*+1/2, *z*+1/2; (iii) −*x*+1, *y*+1/2, −*z*+1/2.
